# Maternal near miss as a predictor of adverse perinatal outcomes: findings from a prospective cohort study in southwestern Uganda

**DOI:** 10.1186/s12884-024-06244-1

**Published:** 2024-01-06

**Authors:** Mark Jjuuko, Henry Mark Lugobe, Richard Migisha, David Collins Agaba, Leevan Tibaijuka, Musa Kayondo, Joseph Ngonzi, Paul Kato Kalyebara, Hamson Kanyesigye

**Affiliations:** 1 Department of Obstetrics and Gynaecology, Mbarara University of Science and Technology, P.O. Box 1410, Mbarara, Uganda; 2 Department of Physiology, Mbarara University of Science and Technology, Mbarara, Uganda

**Keywords:** Maternal near-miss, Adverse perinatal outcomes, Maternal health, Uganda

## Abstract

**Background:**

Despite efforts, Uganda has not met the World Health Organization target of < 12 newborn deaths per 1,000 live births. Severe maternal morbidity or ‘near miss’ is a major contributor to adverse perinatal outcomes, particularly in low-resource settings. However, the specific impact of maternal near miss on perinatal outcomes in Uganda remains insufficiently investigated. We examined the association between maternal near miss and adverse perinatal outcomes at Mbarara Regional Referral Hospital (MRRH) in southwestern Uganda.

**Methods:**

We conducted a prospective cohort study among women admitted for delivery at MRRH’s maternity ward from April 2022 to August 2022. We included mothers at ≥ 28 weeks of gestation with singleton pregnancies, while intrauterine fetal death cases were excluded. For the near-miss group, we consecutively included mothers with any one of the following: antepartum hemorrhage with shock, uterine rupture, hypertensive disorders, coma, and cardiac arrest; those without these complications constituted the non-near-miss group. We followed the mothers until delivery, and their infants until seven days postpartum or death. Adverse perinatal outcomes considered were low birth weight (< 2,500 g), low Apgar score (< 7 at five minutes), intrapartum stillbirths, early neonatal death, or admission to neonatal intensive care unit. Multivariable log-binomial regression was used to determine predictors of adverse perinatal outcomes.

**Results:**

We enrolled 220 participants (55 maternal near misses and 165 non-near misses) with a mean age of 27 ± 5.8 years. Most of the near misses were pregnancies with hypertensive disorders (49%). Maternal near misses had a four-fold (adjusted risk ratio [aRR] = 4.02, 95% CI: 2.32–6.98) increased risk of adverse perinatal outcomes compared to non-near misses. Other predictors of adverse perinatal outcomes were primigravidity (aRR = 1.53, 95%CI: 1.01–2.31), and gestational age < 34 weeks (aRR = 1.81, 95%CI: 1.19–2.77).

**Conclusion:**

Maternal near misses, primigravidity, and preterm pregnancies were independent predictors of adverse perinatal outcomes in this study. We recommend implementing maternal near-miss surveillance as an integral component of comprehensive perinatal care protocols, to improve perinatal outcomes in Uganda and similar low-resource settings. Targeted interventions, including specialized care for women with maternal near misses, particularly primigravidas and those with preterm pregnancies, could mitigate the burden of adverse perinatal outcomes.

## Introduction

The World Health Organization (WHO) defines a maternal near miss as “a woman who nearly died but survived a complication during pregnancy, childbirth, or within 42 days of termination of pregnancy“ [1]. Maternal near miss consist of women who could have died, which points to its severity as well as potential gaps in care; as such, maternal near-miss events serve as crucial indicators of maternal health and the quality of obstetric care provided [2].

According to WHO criteria, the prevalence of near-miss cases in the general population is estimated to be greater than 18.67 per 1,000 live births, worldwide [3]. However, the prevalence varies based on the criteria employed. A systematic review that used disease-specific criteria reported a maternal near-miss prevalence that ranged from 0.80 to 8.23%, while in studies that utilized organ-system-based criteria, the prevalence was found to be between 0.38% and 1.09% [4, 5]. Similarly, in studies employing management-based criteria, the prevalence ranged from 0.01–2.99% [4]. In sub-Saharan Africa, the incidence of maternal near-miss cases ranges from 1.1 to 10% [6]. The maternal near-miss ratio in Uganda was estimated at 8.42 per 1,000 live births in 2017 [7].

In recent years, there has been increasing recognition of the association between maternal near miss and adverse perinatal outcomes [3]. Several studies have shown a significant association between maternal near miss and an increased risk of adverse perinatal outcomes, including preterm birth, low birth weight, neonatal intensive care unit (NICU) admission, and perinatal mortality [3, 7, 8]. Findings from a recent systematic review revealed that women who experienced maternal near-miss events have a 2- to 3-fold higher risk of delivering preterm babies compared to those without such events [3]. Because prematurity is the leading cause of neonatal mortality and morbidity worldwide [9], this underscores the importance of identifying women at risk through maternal near-miss surveillance. Other factors that contribute to poor perinatal outcomes, in addition to maternal near misses, include age, parity, antenatal care attendance, previous history of poor pregnancy outcomes such as stillbirth or neonatal death, referral status, level of education, and mode of delivery [10–14].

In Uganda, perinatal deaths, including stillbirths and early neonatal deaths, are still high, with a perinatal mortality rate estimated at 38 per 1,000 pregnancies [15]; this exceeds the World Health Organization (WHO) target of < 12 stillbirths per 1,000 total births and < 12 newborn deaths per 1,000 live births by more than three-fold [16]. At Mbarara Regional Referral Hospital (MRRH), the incidence of intrapartum stillbirths was 16 per 1,000 deliveries in 2020 [17]. We hypothesized that occurrence of maternal complications was a contributing factor to these adverse perinatal outcomes; this has been documented elsewhere in other regions of the country, especially the central and eastern regions [7]. In Uganda, the most common maternal complications are antepartum hemorrhage, and hypertensive disorders of pregnancy [18, 19]. However, the specific impact of maternal near miss on perinatal outcomes at MRRH in southwestern Uganda is not well documented. It is critical to investigate maternal near-miss clinical outcomes and prognostic implications, in order to develop evidence-based interventions aimed at improving the quality of healthcare and outcomes for women delivering in this low-resource setting, ultimately ensuring the well-being of both mothers and newborns. This study aimed to determine the association between maternal near miss and incidence of adverse perinatal outcomes among women delivering at MRRH, southwestern Uganda.

## Methods

### Study setting and design

This prospective cohort study was conducted at the maternity ward of MRRH from April 2022 to August 2022. The hospital is a public, tertiary hospital in southwestern Uganda and doubles as a teaching hospital for Mbarara University of Science and Technology (MUST). With a bed capacity of approximately 300, MRRH has a catchment population of over three million people across several districts, including Mbarara, Isingiro, Bushenyi, Buhweju, Ibanda, Kiruhura, Mitoma, Ntungamo, Rwamapara, Sheema, and Rubirizi. Additionally, patients from neighboring countries such as Rwanda, Tanzania, and the Democratic Republic of Congo also seek medical care at the hospital. The Obstetrics and Gynecology Department at MRRH handles an average of 9,400 deliveries per year, equivalent to approximately 26 deliveries daily. The department comprised 13 obstetricians, 33 residents, 12 intern doctors, and 19 midwives (with 10 working on any given day), as of 2022.

MRRH serves as a critical referral center, boasting the only functional adult intensive care unit in the region capable of providing life support. Furthermore, it houses a high-risk ward dedicated to managing mothers with complicated pregnancies, making it a crucial destination for those with life-threatening conditions. The hospital also houses a functional neonatal unit specifically designed to address the needs of newborns with complications.

### Study population and eligibility criteria

This prospective cohort study included women with singleton pregnancies who were admitted to Mbarara Regional Referral Hospital (MRRH) for delivery. The participants were divided into two groups: the first group comprised maternal near misses, while the second group consisted of non-near misses. Follow-up was conducted for both groups to assess delivery outcomes, while the infants born to these mothers were monitored from delivery until either seven days postpartum or death, whichever came first.

For the near-miss group, we included mothers who were at 28 weeks of gestation or beyond with singleton pregnancies and any one of the following as adopted from Filippi et al. [20]: antepartum hemorrhage with shock, uterine rupture, eclampsia, severe pre-eclampsia with clinical or laboratory indication for termination of pregnancy to save the woman’s life, chorioamnionitis with clinical signs of shock, coma, as well as cardiac arrest in whom a decision to deliver had been made by the clinical healthcare team.

For the non-near-miss group, we included mothers who were at 28 weeks of gestation or beyond with singleton pregnancies not fulfilling any of the near-miss criteria in whom a decision to deliver had been made by the clinical healthcare team. For both groups, we excluded mothers with intrauterine fetal death at admission.

### Sample size estimation and sampling

The sample size was calculated using Open Epi software [21], specifically employing the sample size determination for cohort studies. The calculations were based on the following assumptions: a 95% confidence level, 80% power, an exposed (maternal near miss) to non-exposed (non-near miss) ratio of 1:3, a 22.2% occurrence of the outcome (low Apgar score) in the exposed group, and a 6% occurrence of the outcome in the unexposed group. These assumptions were derived from a previous study conducted in Nigeria [22]. Considering a 10% loss to follow-up rate, the final sample size required for the study was determined to be 55 participants in the exposed group and 165 participants in the non-exposed group, resulting in a total of 220 participants.

Participants identified as maternal near misses were consecutively sampled until the desired sample size was reached. For each maternal near miss identified, three non-near misses who were admitted for delivery following the identification of the maternal near miss, as recorded in the admission register, were consecutively recruited until the required sample size was attained.

### Study variables

The primary exposure variable was maternal near miss. This was measured using Filippi criteria [20]. A mother was classified as a near miss if she fulfilled at least one of these criteria. The specific definitions used for various conditions were as follows:

- Shock: Systolic blood pressure < 90 mmHg or diastolic blood pressure < 60 mmHg.
- Severe pre-eclampsia: Presence of preeclampsia with elevated serum transaminase concentration, severe persistent right upper quadrant pain or epigastric pain, thrombocytopenia, increased serum creatinine, and hypertension.
- Hypertension: Blood pressure ≥ 140 mmHg (systolic) or ≥ 90 mmHg (diastolic).
- Eclampsia: Hypertension associated with fits, with or without proteinuria.
- Proteinuria: Protein ≥ 2 + on a dipstick.
- Cardiac arrest: Loss of consciousness and absence of pulse or heartbeat requiring cardiopulmonary resuscitation.
- Coma: Glasgow Coma Scale < 9.
- Uterine rupture: Complete rupture of the uterus, including the peritoneum.
- Chorioamnionitis: Presence of foul-smelling liquor.

The study also considered several independent variables, including demographic characteristics (e.g., age, education level), obstetric characteristics and medical characteristics such as gravidity, gestational age at birth (determined from the last normal menstrual period [LNMP] or first-trimester ultrasound scan where the mother did not recall her LNMP), antenatal care (ANC) visits, inter-delivery interval, HIV serostatus (documented result within the past three months), decision-to-delivery time, mode of delivery, obstructed labor, and premature rupture of membranes.

The outcome variable of interest was adverse perinatal outcomes, This was a composite outcome which included any one of the following: low birth weight (< 2500 g), low Apgar score (< 7 at the fifth minute of life), intrapartum stillbirth, early neonatal death (within seven days of life), and admission to the NICU within seven days of life.

### Recruitment and follow-up of participants

At admission, charts of mothers were screened for inclusion. Accordingly, maternal near misses and non-near misses were identified by the research assistants. Mothers for whom a decision to deliver had been made provided informed consent. Management of all study participants was done by the clinical care team and the research team did not interfere with the routine care. Pregnant mothers were monitored for delivery outcomes. When delivery resulted in a still birth then the follow-up concluded. Babies born alive were followed up on each day up to day seven, following delivery or until they died whichever came first. For mother-baby pairs that had been discharged before seven days postdelivery, a phone call was made to the mother on a daily basis to inquire about the status of the baby (i.e., whether the baby was alive or readmitted or dead). Mothers who had no phones were contacted through their kindred who owned phones. The research assistants went to NICU to confirm admission for those babies who had been referred to NICU and their admission diagnoses. The research assistants, all of whom were experienced midwives, received comprehensive training from the principal investigator. This training equipped them with the expertise to identify maternal near misses, assess adverse perinatal outcomes, and execute the study procedures.

### Data management and analysis

Data were cleaned, coded, and entered into the RED-CAP software [23]. Subsequently, the dataset was exported to STATA 15 (StataCorp, College Station, Texas, USA) for further analysis. The first step in our analysis involved describing the baseline demographic, obstetric, and medical characteristics of the participants using proportions. We then compared the proportions of adverse perinatal outcomes between two groups, namely maternal near misses and non-maternal near misses, using the Chi-square test.

To determine the association between maternal near miss and adverse perinatal outcomes for the study participants, we performed both crude and adjusted risk ratio calculations. For the univariable analysis, we employed log-binomial regression analysis. In the multivariable analysis, we utilized the same regression analysis, adjusting for confounding variables. In the final multivariable model, we included all exposure variables with a *p*-value < 0.2 at univariable analysis, as well as age, based on biological plausibility. The primary exposure variable was maternal near miss, and its effect on adverse perinatal outcomes was assessed, while adjusting for other confounding variables. To assess collinearity in our multivariable model, we employed the variance inflation factor (VIF) method, considering VIF > 5 as indicative of collinearity. The predictors for adverse perinatal outcomes in the multivariable model were determined based on variables with a *p*-value < 0.05, indicating statistical significance.

## Results

**Fig. 1:**
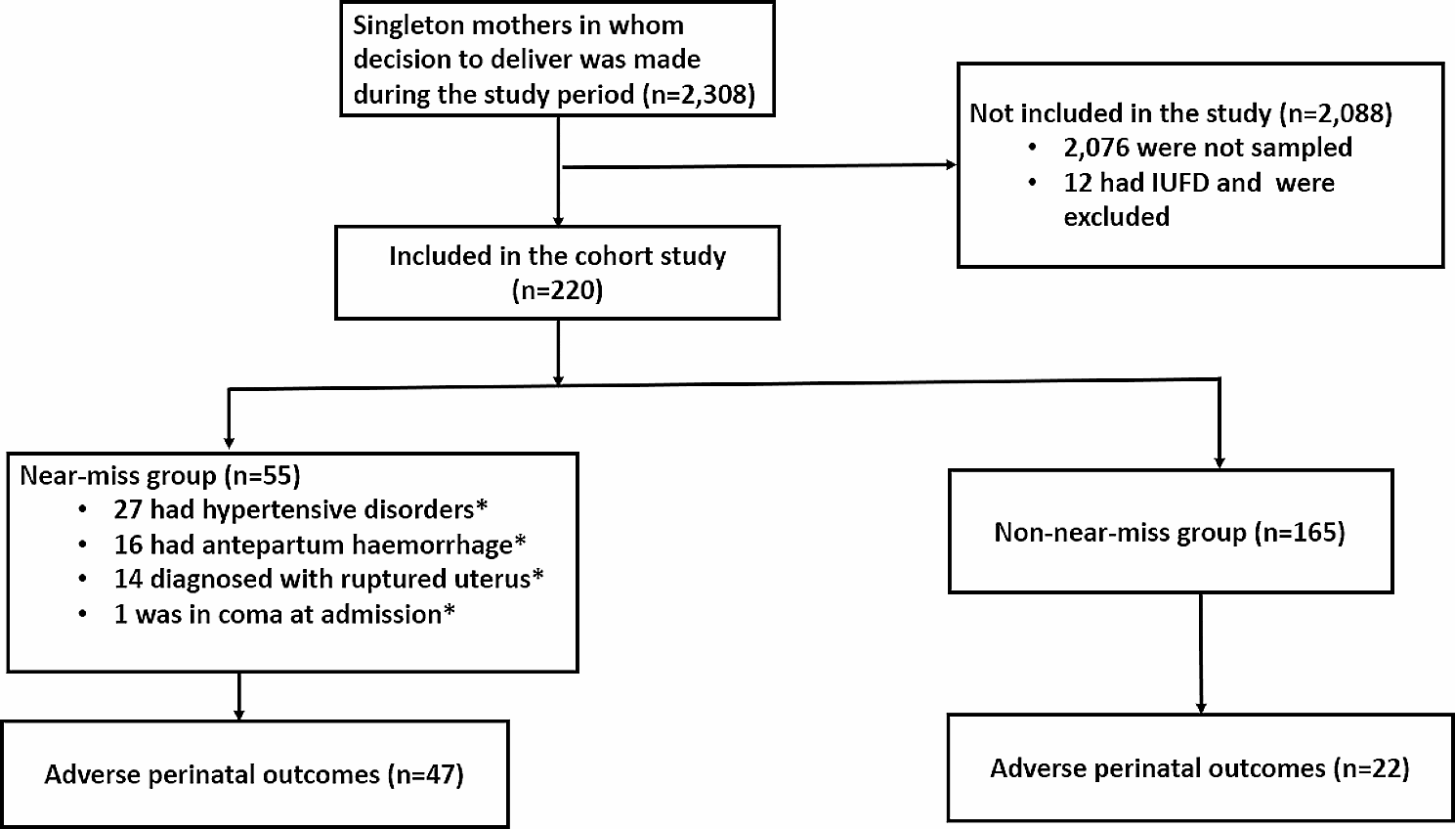
Flow chart showing recruitment, exclusion, and follow-up of participants, at Mbarara Regional Referral Hospital, April – August, 2022; *types of maternal complications were not mutually exclusive; IUFD: Intra uterine fetal death

Of the 2,308 mothers screened for inclusion into the study from April 2022 to August 2022, a total of 220 were recruited into the study. Among these, 55 were maternal near misses and 165 non-near misses. We excluded 12 mothers because they had intrauterine fetal death at admission. Among the 55 near misses, most common complication was hypertensive disorders, accounting for nearly half of the cases (49%; *n* = 27), followed by antepartum haemorrhage (29%; *n* = 16) ruptured uterus (25%; *n* = 14) and coma (1.8% *n* = 1) (Fig. 1).

### Characteristics of study participants

The mean age was 27 ± 5.8 years; the majority of participants fell within the 20–34 years category (79.5%). The highest proportion had at least a secondary education (53.6%). Regarding gravidity, half of the participants had 2–4 pregnancies (50.0%). The vast majority of participants attended antenatal care (ANC) (99.1%); there was a significant difference (*p* < 0.001) in the number of ANC visits, with a higher proportion of near-miss cases having less than four visits (51.9%), compared to the non-near miss cases (20.1%) who predominantly had four or more visits (79.9%) (Table 1).

**Table 1:** Participants’ characteristics in near-miss and non-near-miss groups at Mbarara Regional Referral Hospital, Uganda, April–August, 2022

Variable	Near-miss (*n* = 55), n (%)	Non-near miss (*n* = 165), (n %)	*P* value
Age* category (years)			0.536
< 20	4 (7.3)	21 (12.7)	
20–34	45 (81.8)	130 (78.8)	
≥ 35	6 (10.9)	14 (8.5)	
**Education level**			0.001
<Secondary	36 (65.5)	66 (40.0)	
≥Secondary	19 (34.5)	99 (60.0)	
**Gravidity**			0.048
1	12 (21.8)	66 (40.0)	
2–4	35 (63.6)	76 (46.1)	
≥ 5	9 (16.4)	23 (13.9)	
**History of stillbirth**‡	5 (11.6)	7 (7.1)	0.370
**Attended ANC**	54 (98.2)	164 (99.4)	0.412
**Number of ANC visits**†			< 0.001
≥ 4	26 (48.1)	131 (79.9)	
< 4	28 (51.9)	33 (20.1)	
**Duration since last delivery**‡			0.555
< 33 months	22 (51.2)	46 (46.5)	
33–68 months	17 (39.5)	37 (37.4)	
> 68 months	4 (9.3)	16 (16.2)	
**Gestational age**			< 0.001
28–33 weeks	22 (40.0)	3 (1.8)	
34–36 weeks	10 (18.2)	4 (2.4)	
37–41 weeks	21 (38.2)	155 (93.9)	
≥ 42 weeks	2 (3.6)	3 (1.8)	
**Prelabour rupture of membranes**¶	4 (10.8)	10 (6.9)	0.418
**Obstructed labour**¶	3 (7.7)	7 (4.8)	0.477
**Referred from another facility**	40 (72.7)	51 (30.9)	< 0.001
**Cadre of birth attendant**			< 0.001
Midwife	4 (7.3)	70 (42.4)	
Intern doctor	1 (1.8)	14 (8.5)	
Resident or specialist	50 (90.9)	81 (49.1)	
**Mode of delivery**			< 0.001
Spontaneous vertex	9 (16.4)	79 (47.9)	
Assisted vaginal	2 (3.6)	4 (2.4)	
Caesarean	36 (65.4)	82 (49.7)	
Laparotomy	8 (14.6)	0 (0.0)	
**Decision to delivery time§**			0.023
≤ 30 min	8 (18.2)	4 (4.9)	
31–60 min	14 (31.8)	20 (24.4)	
> 60 min	22 (50.0)	58 (70.7)	
**HIV positive**	0 (0.0)	20 (12.1)	0.005

The majority of participants had a gestation period between 37 and 41 weeks (80.0%); the distribution of gestational age between the near-miss and non-near-miss groups was significantly different (*p* < 0.001). Various cadres of healthcare workers conducted deliveries, with midwives attending to 33.6% of the participants, intern doctors to 6.8%, and residents or specialists to 59.6%. Regarding the mode of delivery, a significant difference was observed, with near misses having higher proportions of caesarean deliveries (65.4%) and laparotomies (14.6%) compared to non-near misses (49.7% and 0.0% respectively). In contrast, non-near misses had a higher proportion of spontaneous vertex deliveries (47.9%) compared to near-miss cases (16.4%) (*p* < 0.001).

The distribution of HIV status differed significantly between the near-miss and non-near miss groups (*p* < 0.005). Among the participants, 9.1% of the total cohort were HIV positive. None of the participants in the near-miss group were HIV positive, while 12.1% of the participants in the non-near miss group were positive for HIV.

### Incidence of adverse outcomes in the study cohort

Overall, the near-miss group had a significantly higher incidence of any adverse outcome (83.6%) compared to the non-near-miss group (13.9%). There were significant differences between the two groups for all adverse outcomes assessed (*p* < 0.001) (Table 2).

**Table 2:** Comparison of adverse perinatal outcomes between near-miss and non-near miss groups in the cohort at Mbarara Regional Referral Hospital, Uganda, April–August, 2022

Adverse outcome	Near-miss (*n* = 55), n (%)	Non-near miss (*n* = 165), (n %)	*P* value
Low Birth weight (< 2.5 kg)	31 (56.4)	4 (2.4)	< 0.001
Low APGAR score (< 7 at 5 min)	24 (43.6)	8 (4.9)	< 0.001
Intrapartum stillbirth	22 (40.0)	6 (3.6)	< 0.001
Baby died before the end of the first week of life	28 (50.9)	9 (5.5)	< 0.001
Baby admitted to neonatal intensive care unit.	15 (27.3)	11 (6.7)	< 0.001
Any adverse outcome	46 (83.6)	23 (13.9)	< 0.001

Among the total cohort, 15.9% of participants had low birth weight (< 2.5 kg). However, within the near-miss group, there was a high incidence of low birth weight (56.4%), compared to the non-near-miss group (2.4%). Similarly, 14.6% of the total cohort had a low APGAR score (< 7 at 5 min); in the near-miss group, this proportion was considerably higher at 43.6%, while it was much lower at 4.9% in the non-near-miss group.

Intrapartum stillbirths were observed in 12.7% of the total cohort. Among near-miss cases, the rate was 40.0%, whereas in the non-near miss group, it was 3.6%. The proportion of neonates who died before the end of the first week of life was 16.8% in the total cohort. However, within the near-miss group, it was high (50.9%), while it was substantially lower at 5.5% in the non-near-miss group (Table 2).

### Association between maternal near miss, demographic and medical exposure variables, and adverse perinatal outcomes

In the multivariable analysis (Table 3), maternal near misses were found to be independently associated with adverse perinatal outcomes, with a four-fold higher risk compared to non-near misses (aRR = 4.02, 95%CI: 2.32–6.98, *p* < 0.001). Additionally, gestational age and gravidity were also identified as independent factors associated with adverse perinatal outcomes. Participants with gestational ages ranging from 28 to 33 weeks had approximately 1.8 times (aRR = 1.81, 95%CI: 1.19–2.77, *p* = 0.006) higher risk of experiencing adverse perinatal outcomes compared to those at term (37–41 weeks). Similarly, primigravidas had a 1.5-fold (aRR = 1.53, 95%CI: 1.01–2.31, *p* = 0.044) higher risk of adverse perinatal outcomes compared to participants with a gravidity of two to four.

**Table 3:** Association between maternal near miss, demographic and clinical exposure variables and adverse perinatal outcomes at Mbarara Regional Referral Hospital, Uganda, April–August, 2022

Variable	Adverse outcome		Unadjusted analysis		Multivariable analysis	
	Yes (*n* = 69), n (%)	No (*n* = 151), n (%)	cRR (95%CI)	*P*-value	aRR (95%CI)	*P*-value
**Maternal near-miss**						
No	23 (33.3)	142 (94.0)	Ref		Ref	
Yes	46 (66.7)	9 (6.0)	6.41 (4.28–9.60)	< 0.001	4.02 (2.32–6.98)	< 0.001**
**Age category (years)**						
< 20	6 (8.7)	19 (12.6)	0.76 (0.37–1.59)	0.470	0.80 (0.43–1.48)	0.478
20–34	55 (79.7)	120 (79.5)	Ref		Ref	
≥ 35	8 (11.6)	12 (7.9)	1.27 (0.71–2.27)	0.415	0.88 (0.40–1.94)	0.747
**Referral status**						
Referred in	45 (65.2)	46 (30.5)	2.66 (1.75–4.03)	< 0.001	1.35 (0.92–1.97)	0.122
Not referred in	24 (34.8)	105 (69.5)	Ref		Ref	
**Education level**						
<Secondary	39 (56.5)	63 (41.7)	1.50 (1.01–2.23)	0.043	0.86 (0.60–1.24)	0.413
≥Secondary	30 (43.5)	88 (58.3)	Ref		Ref	
**Gravidity**						
1	21 (30.4)	57 (37.8)	0.90 (0.56–1.43)	0.647	1.53 (1.01–2.31)	0.044**
1–4	33 (47.8)	77 (51.0)	Ref		Ref	
≥ 5	15 (21.7)	17 (11.3)	1.56 (0.98–2.49)	0.061	1.71 (0.97–3.01)	0.066
**Number of ANC visits**						
≥ 4	39 (57.4)	118 (78.7)	Ref		Ref	
< 4	29 (42.7)	32 (21.3)	1.91 (1.31–2.80)	0.001	0.81 (0.55–1.19)	0.282
**Gestational age**						
28–33 weeks	23 (33.3)	2 (1.3)	4.76 (3.45–6.58)	< 0.001	1.81 (1.19–2.77)	0.006**
34–36 weeks	9 (13.0)	5 (3.3)	3.33 (2.03–5.45)	< 0.001	1.27 (0.76–2.15)	0.362
37–41 weeks	34 (49.3)	142 (94.1)	Ref		Ref	
≥ 42 weeks	3 (4.4)	2 (1.3)	3.11 (1.43–6.75)	0.004	1.46 (0.85–2.50)	0.173
**Cadre of birth attendant**						
Midwife	8 (11.6)	66 (43.7)	Ref		Ref	
Intern doctor	3 (4.4)	12 (7.9)	2.11 (0.62–7.23)	0.234	2.14 (0.62–7.40)	0.229
Resident or specialist	58 (84.1)	73 (48.3)	4.76 (2.29–9.88)	< 0.001	1.82 (0.73–4.52)	0.200
**Mode of delivery**						
Spontaneous vertex	13 (18.8)	75 (49.7)	Ref		Ref	
Assisted vaginal	3 (4.4)	3 (2.0)	3.38 (1.32–8.70)	0.011	1.60 (0.75–3.41)	0.224
Caesarean	46 (66.7)	72 (47.7)	2.64 (1.52–4.58)	0.001	1.24 (0.72–2.14)	0.436
Laparotomy	7 (10.1)	1 (0.6)	5.92 (3.36–10.43)	< 0.001	1.54 (0.75–3.20)	0.240
Decision to delivery time*						
≤ 30 min	8 (15.1)	4 (5.4)	Ref		–	–
31–60 min	16 (30.2)	18 (24.7)	0.71 (0.41–1.21)	0.203		
> 60 min	29 (54.7)	51 (69.9)	0.54 (0.33–0.89)	0.016		
HIV status*						
Positive	1 (1.5)	19 (12.6)	0.15 (0.02-1.00)	0.050	–	–
Negative	68 (98.5)	132 (87.4)	Ref			

RR: Risk ratio; cRR: crude risk ratio; aRR: adjusted risk ratio; CI: Confidence interval; ANC: Antenatal care

*Eliminated from the multivariable model because of collinearity

**Independent predictors for adverse perinatal outcomes (*p*-value < 0.005)

## Discussion

This prospective cohort study examined the association between maternal near miss and adverse perinatal outcomes at a tertiary hospital in a low-resource setting in southwestern Uganda. The results showed that maternal near misses were independently associated with a four-fold higher risk of adverse perinatal outcomes compared to non-near misses. Additionally, gestational age and gravidity were identified as independent predictors of adverse perinatal outcomes, with primigravidas and mothers with preterm pregnancies (28 to 33 weeks) having approximately a two-fold increased risk.

In the current study, maternal near miss was independently associated with increased risk for adverse perinatal outcomes. This is consistent with previous studies conducted in sub-Saharan Africa [10, 12, 22] and Indonesia [11]. Maternal complications leading to maternal near miss, such as severe pre-eclampsia, antepartum hemorrhage, and ruptured uterus, compromise placental perfusion and disrupt the exchange of nutrients between mother and fetus. This results in fetal compromise, leading to a range of adverse outcomes including premature termination of pregnancy, stillbirth, intrauterine growth restriction, low birth weight, low Apgar scores, and increased early neonatal morbidity and mortality [24]. On the basis of our findings, we recommend that maternal near-miss audits be implemented as a valuable tool for identifying healthcare gaps and improving perinatal outcomes among maternal near-misses in Uganda and other similar low-resource settings. As previously demonstrated, such audits enable healthcare facilities to gain insights into areas for improvement and develop targeted interventions to address identified healthcare gaps [25].

In addition to maternal near miss, other significant independent factors associated with adverse perinatal outcomes were primigravidity and gestational age of < 34 weeks. According to some studies, primigravidity is associated with adverse perinatal outcomes [18, 26, 27]; however, others have shown no such association [13, 28]. Primigravidas tend to be younger than multigravidas which influences their health-seeking behavior; younger patients tend to be less aware of the importance of antenatal care which results in fewer visits and late booking visits, hence contributing to delays in detecting pregnancy complications [29, 30]. Furthermore, hypertensive disorders tend to be more prevalent among the primigravidas than multigravidas which may increase the risk of adverse perinatal outcomes in this group [30, 31]. Additionally, primigravidas tend to be more prone than multigravidas to malaria which may compromise placental perfusion resulting in an increased risk of adverse perinatal outcomes such as low birth weight and stillbirth [32, 33]. Given the association between primigravidity and adverse perinatal outcomes, deliberate efforts should be made to improve care for primigravidas, including promoting awareness of the importance of antenatal care, facilitating early and regular antenatal visits, and addressing delays in detecting pregnancy complications in this population.

Being preterm (gestational age < 34 weeks) was associated with an increased risk of adverse perinatal outcomes in the current study. This finding is consistent with a previous prospective cohort study conducted at the same hospital, among mothers with hypertensive disorders [19]. Preterm birth remains the leading cause of neonatal mortality worldwide, contributing to approximately three out of every ten neonatal deaths [9]. The underlying reasons for these adverse perinatal outcomes are primarily complications associated with prematurity, including respiratory distress syndrome, hypothermia and hypoglycemia. Furthermore, hyperbilirubinemia, necrotizing enterocolitis, and sepsis further contribute to the burden of adverse outcomes in extremely premature infants [34, 35]. Considering the increased risk of adverse perinatal outcomes associated with extreme prematurity, we recommend a strong emphasis on preterm birth prevention and management, including improving the detection and management of complications related to prematurity.

This study had various strengths: there was no loss to follow-up, and the study considered various adverse outcomes consisting of intrapartum stillbirth, admission to NICU, early neonatal death, low birth weight and low Apgar score; this enriches the depth of our insights. Furthermore, the maternal near-miss criteria employed in this study makes it readily replicable in similar low-resource settings. This not only enhances the generalizability of the findings but also contributes to the potential utility of the study framework in addressing maternal health concerns in comparable contexts.

A noteworthy limitation of our study is that we did not assess the long-term maternal and neonatal consequences resulting from near-miss events. To provide a more comprehensive understanding of the impact of maternal near miss, we recommend that future studies focus on investigating the long-term neonatal and maternal outcomes associated with near-miss events. It is important to acknowledge that near-miss events encompass occurrences within 42 days of pregnancy termination; however, our study describes mainly antepartum and intrapartum near misses.

## Conclusion

Our study provides compelling evidence that maternal near misses are associated with a higher risk of adverse perinatal outcomes compared to non-near misses. Additionally, our analysis identified primigravidity and being preterm (gestational age below 34 weeks), as independent predictors of adverse perinatal outcomes among women delivering at this low-resource setting in southwestern Uganda. These findings underscore the importance of targeted interventions and specialized care for maternal near misses, such as those with hypertensive disorders, uterine rupture, as well as primigravidas and those with preterm pregnancies, to mitigate the risk and burden of adverse perinatal outcomes. Implementing maternal near-miss surveillance as an integral component of comprehensive perinatal care protocols, could improve perinatal outcomes in Uganda and other similar low-resource settings. We recommend further studies to assess the feasibility, acceptability and cost-effectiveness of integrating maternal near-miss surveillance into routine perinatal care protocols in our setting.

## Data Availability

The datasets generated and analysed for this study are available from the corresponding author, upon request.

## Abbreviations

ANC: Antenatal care
APH: Antepartum hemorrhage
aRR: Adjusted risk ratio
cRR: Crude risk ratio
HIV: Human Immunodeficiency Virus
IUFD: Intrauterine fetal death
MRRH: Mbarara Regional Referral Hospital
MUST: Mbarara University of Science and Technology
NICU: Neonatal intensive care unit
WHO: World Health Organization
